# Nanoscale Sub-Compartmentalization of the Dendritic Spine Compartment

**DOI:** 10.3390/biom11111697

**Published:** 2021-11-15

**Authors:** Ana Sofía Vallés, Francisco J. Barrantes

**Affiliations:** 1 Instituto de Investigaciones Bioquímicas de Bahía Blanca (UNS-CONICET), Bahía Blanca 8000, Argentina; svalles@criba.edu.ar; 2 Laboratory of Molecular Neurobiology, Institute of Biomedical Research (BIOMED), UCA-CONICET, Av. Alicia Moreau de Justo 1600, Buenos Aires C1107AFF, Argentina

**Keywords:** dendritic spine, plasma membrane, membrane domains, nanodomains, neurotransmitter receptors, cannabinoids, acetylcholine receptor, glutamatergic receptor, NMDAR, AMPAR, cannabinoid receptor, GPCR

## Abstract

Compartmentalization of the membrane is essential for cells to perform highly specific tasks and spatially constrained biochemical functions in topographically defined areas. These membrane lateral heterogeneities range from nanoscopic dimensions, often involving only a few molecular constituents, to micron-sized mesoscopic domains resulting from the coalescence of nanodomains. Short-lived domains lasting for a few milliseconds coexist with more stable platforms lasting from minutes to days. This panoply of lateral domains subserves the great variety of demands of cell physiology, particularly high for those implicated in signaling. The dendritic spine, a subcellular structure of neurons at the receiving (postsynaptic) end of central nervous system excitatory synapses, exploits this compartmentalization principle. In its most frequent adult morphology, the mushroom-shaped spine harbors neurotransmitter receptors, enzymes, and scaffolding proteins tightly packed in a volume of a few femtoliters. In addition to constituting a mesoscopic lateral heterogeneity of the dendritic arborization, the dendritic spine postsynaptic membrane is further compartmentalized into spatially delimited nanodomains that execute separate functions in the synapse. This review discusses the functional relevance of compartmentalization and nanodomain organization in synaptic transmission and plasticity and exemplifies the importance of this parcelization in various neurotransmitter signaling systems operating at dendritic spines, using two fast ligand-gated ionotropic receptors, the nicotinic acetylcholine receptor and the glutamatergic receptor, and a second-messenger G-protein coupled receptor, the cannabinoid receptor, as paradigmatic examples.

## 1. Introduction

The plasma membrane is currently envisaged as a highly heterogeneous and compartmentalized two-dimensional lattice. Convergent biochemical and biophysical tools have provided experimental support to this notion (see reviews in [1,2,3]). The finding of coexisting lipid domains with distinct physicochemical properties (liquid-ordered and liquid-disordered phases, respectively) observed in biomimetic synthetic lipid membranes inspired the “lipid raft” hypothesis, which purported that similar co-occurrence of phases also occurred in natural cell membranes, whereby rafts corresponded to cholesterol- and sphingolipid-rich liquid-ordered domains [4,5]. The functional implications of the hypothesis were vast: the early proposal that these lipid domains served as signaling platforms was an appealing idea [6] that soon extended to various branches of cell biology. Intracellular signaling pathways require adequate membrane organization, and the concept of membrane compartments provided the substrate to explain how dynamic processes could occur separately and simultaneously in spatio-temporally separateddiscrete domains.

In the central nervous system (CNS), most of the excitatory synapses occur on a specialized subcellular formation in neuronal arborizations, so-called dendritic spines. These spines constitute highly differentiated subcellular compartments of the receiving (postsynaptic) neuron, concentrating in a very small volume an abundant collection of neurotransmitter receptors, enzymes, scaffolding proteins, and cytoskeletal elements. Various neurotransmitter receptor systems harbored in the spine postsynaptic membrane have been shown to be associated with liquid-ordered, raft-type lipid domains [7,8,9,10]. These lipid domains not only harbor the receptor proteins but also recruit them from extra-synaptic regions; once receptors reach the active zone of the spine, the more rigid liquid-ordered domains contribute to reducing their lateral mobility [11,12]. These neurotransmitter receptor-containing lipid assemblies have dimensions in the order of nanometers, hence the designated term “nanodomains”. The surface of the mature spine, a morphological entity of ≤1 μm diameter, is thus sub-compartmentalized into much smaller parcels that contribute to the lateral heterogeneity of the postsynaptic membrane. This description of the dendritic spine is, however, fragmentary inasmuch it does not contemplate its highly dynamic nature, especially of some of its patchy nanodomains. Thus both stable and dynamic platforms coexist in the spine postsynaptic complex, and the dynamic range of the two combined is quite ample, from a few milliseconds to days [13], providing spatio-temporal stability to some of its components while allowing fast turnover and agile redistribution in others.

Pre- and postsynaptic membranes share the property of organizing their constituents in the form of nanodomains characterized by high molecular densities and transient duration. A characteristic feature of this organizational principle is that it is asymmetric (see review in [14]), in accordance with the vectorial nature of the synapse.

In this review, we focus on the structural and functional roles played by lipids and proteins in such nanodomains, discussing the significance of compartmentalization for efficient synaptic signaling. Examples are provided on how the glutamatergic and the cholinergic neurotransmitter receptors, two well characterized fast operating ligand-gated ion channels, and the endocannabinoid system, a typical metabotropic second-messenger G-protein coupled receptor (GPCR), tend to localize in clustered structures, the cholinergic and the cannabinoid receptors often overlapping anatomically, and share common roles in several physiological and pathological processes that exploit and rely on membrane nanodomains.

## 2. Dendritic Spines, Discrete Subcellular Compartment of the Neuronal Membrane

Dendritic spines constitute fundamental units of information endowed with processing synaptic (mostly excitatory) chemical neurotransmission in the mammalian brain. Spines compartmentalize biochemical and electrical signals, thus modulating the functional properties of synapses. Based on the relative length of their neck and the diameter of their head, dendritic spines have been arbitrarily classified into five categories: mushroom, thin, stubby, filopodia, and bifurcation- or cup-shaped spines [15]. However, this represents a fragmentary, anatomically static view of the dendritic spine: in fact, they also have a dynamic dimension, allowing them to modify their size and shape within seconds to minutes and undergo more lasting changes in the time scale of hours to days. In mature neurons spine motility declines [13]. Spine head volumes range from 0.01 to 1 μm^3^, and spine necks vary between 50 and 500 nm in diameter and up to 3 μm in length [16,17,18]. The size of the spine head has been shown to correlate with the size of the postsynaptic density (PSD) a characteristic morphological differentiation of the postsynapse [16,17,18], and with the amplitude of the excitatory postsynaptic current (EPSC) [19,20].

**Figure 1:**
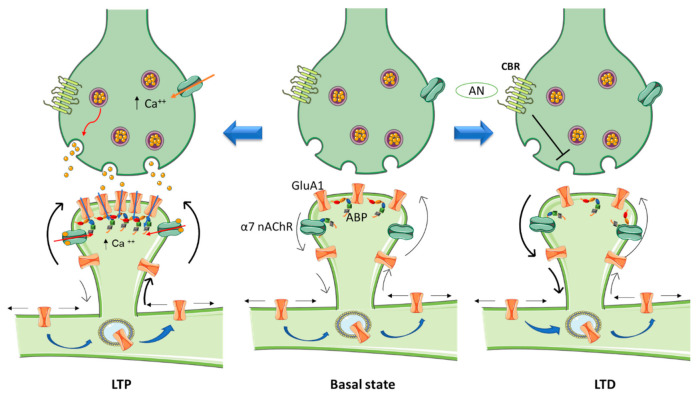
Schematic diagram highlighting the role of nanoscale sub-compartmentalization of the dendritic spine in synaptic plasticity. The “basal” state can be depicted as a homeostatic equilibrium between synthesis, lateral diffusion, internalization, degradation, and recycling of neurotransmitter receptors at the dendritic spine. Activation of α7 nicotinic acetylcholine receptor (α7 nAChR) in the hippocampus by agonist on postsynaptic sites promotes LTP (left) by depolarizing the spine which induces glutamatergic GluA1 receptors to cluster at the PSD. This incorporation of GluA1 receptor molecules further contributes to calcium entry, thus strengthening synaptic transmission. The opposite phenomenon (LTD, right) is induced by activation of presynaptic cannabinoid receptors (CBRs): neurotransmitter release is inhibited, thereby weakening synaptic transmission, and GluA1 receptors are depopulated from the PSD. ABP: actin binding proteins; AN: anandamine.

One of the most important functions of the brain is enabling the neural activity generated by an experience to modify neural circuit functionality in a process referred to as synaptic plasticity. This property of individual synapses affects higher brain functions like thought, feeling, and behavior [21]. Morphological correlates of synaptic plasticity at the dendritic spine level have been described, involving the remodeling of the PSD to regulate the number of intervening receptors and scaffolding proteins. The so-called long-term potentiation (LTP) and long-term depression (LTD), two mechanisms that have been thoroughly studied in the mammalian hippocampus, are induced by recent patterns of activity that strengthen or depress synapse activity, respectively [22] (Figure 1).

**Figure 2:**
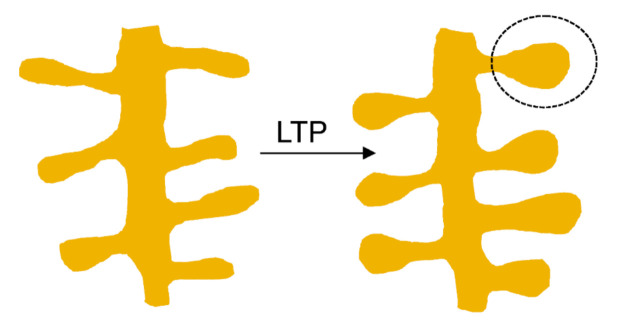
Schematic depiction of the enlargement and acquisition of mature mushroom-like shape of dendritic spines following induction of long-term potentiation (LTP) during synaptic plasticity.

These functional readouts have straightforward morphological correlates: spine head enlargement in LTP [23,24] and conversely, shrinkage of the spine head and increased spine loss, as observed in hippocampal synapses after electrical induction of LTD [25,26]. The width of the spine neck has been associated with the degree of compartmentalization of synaptic signals [27], and its length with the modulation of synaptic membrane tension [28]. Upon induction of LTP, spine necks become shorter and wider [27] (Figure 2).

**Figure 3:**
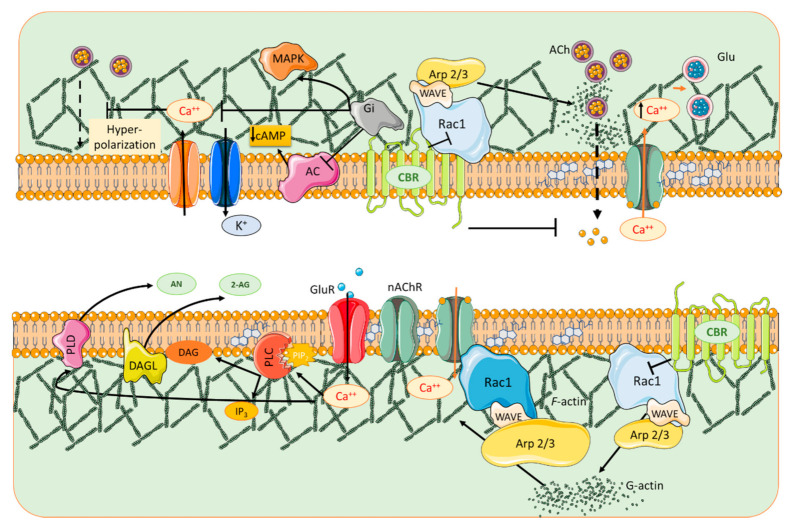
Schematic diagram of a vomposite synapse summarizing the pre- and postsynaptic components participating in synaptic signaling dissected in this review. Glutamatergic (Glu) and cholinergic (α7 nAChR) activation promotes Ca^2+^ entry into the dendritic spine, inducing endocannabinoid (eCB) synthesis through hydrolysis of lipid precursors from the cell membrane. Cannabinoid receptor (CBR) activation in dendritic spines inhibits Rac1/WAVE/Arp 2/3 and limits the conversion of G actin to F-actin, whereas α7 nAChR promotes formation and maturation of dendritic spines through F-actin stability by activating the Rac1/WAVE/Arp2/3 signaling pathway. Phospholipase C (PLC) converts PIP2 into diacylglycerol (DAG) and in turn DAG lipase (DAGL) generates the eCB 2-AG. In parallel, phospholipase D (PLD) converts N-arachidonoyl phosphatidylethanolamine into the eCB AN. 2-AG and AN are liberated into the synaptic cleft and activate CBRs in the presynaptic compartment. Upon activation, CBRs stimulate Gi-protein and inhibit AC activity, membrane hyperpolarization ensues after the modulation of K^+^ and Ca^2+^ channels that inhibit neurotransmitter release from the presynaptic compartment. Finally, the mitogen-activated protein kinase (MAPK) pathway is stimulated. AC: adenyl cyclase; cAMP: cyclic AMP; ACh; acetylcholine; 2-AG, 2-arachidonyl glycerol.

Compartmentalization of dendritic spines spatially constrains the diffusion of second messenger molecules to small volumes [29], thus circumscribing biochemical cascades and regulating information processing in individual synapses to smaller regions thereof. For example, presynaptic stimulation can elicit Ca^+2^ transient currents that are confined to hot spots of activity in spines [30,31]. The biochemical compartmentalization of the membrane requires membrane-bound enzymes, lipids and proteins to be organized into delimited regions of varying size and composition [32]. In addition to the delimitation in space, membrane domains vary in duration, thereby intervening in the timing of biochemical events at the synapse. This spatio-temporal organization extends to the coordination between pre- and postsynaptic components of the synaptic micro-cosmos (Figure 3).

## 3. Cannabinoids and Cannabinoid Receptors

The endocannabinoid system (ECS) is involved in almost all aspects of mammalian physiology and pathology. The ECS consists of genes encoding cannabinoid receptors (CBRs), endogenous cannabinoid ligands (endocannabinoids, eCBs), and the enzymes involved in their synthesis and degradation. Cannabinoids can be grouped into three major subclasses: phytocannabinoids, which are extracted from plants; eCBs, produced by live organisms, and synthetic cannabinoids that share some or all of the physiological properties of either phytocannabinoids and/or eCBs.

eCBs are lipidic, arachidonic acid-containing neurotransmitters (or messengers) that are synthesized de novo by phospholipase action after hydrolyzing the lipid precursors from the cellular membrane [33]. They are produced on demand when needed and are not usually stored in vesicles like classical neurotransmitters [34]. The synthesis of eCBs takes place upon postsynaptic depolarization through Ca^2+^ influx and activation of Gq-protein. The heterotrimeric G protein Gq stimulates eCB production through phospholipase C and D activation. After being synthesized in postsynaptic neurons, eCBs are released to act on CBRs expressed in presynaptic and/or nearby neurons.

Arachidonoyl ethanolamine (anandamine, AN), the first eCB discovered [35], is an endogenous lipid neurotransmitter derived from the polyunsaturated fatty acid arachidonic acid. AN is more soluble in aqueous media than arachidonic acid. AN acts as a partial agonist of CBR1 and CBR2 and as a full agonist of the vanilloid receptor 1 (VR1). The other eCB that acts as a full agonist of CBR1 and CBR2 is 2-arachidonyl glycerol (2-AG). Additionally, eCBs can activate other “non-CB” receptors, such as G protein-coupled receptor 55 (GPR55), peroxisome proliferator-activated receptors (PPARs) [36]. Although CBRs are mostly found on plasma membranes, their localization at intracellular compartments such as the endoplasmic reticulum (ER), endosomes, lysosomes, mitochondria and nuclei has also been demonstrated [37].

The two cannabinoid receptors identified to date have been termed CBR1 and CBR2. They share 44% amino acid sequence homology [38,39]. CBR1 constitutes the most abundant G-protein coupled receptor (GPCR) in the CNS [40], whereas CBR2 is localized preferentially in immune cells and peripheral tissues [39] and to a lesser extent in the brainstem, cortex, hippocampus and cerebellar neurons and microglia [41,42]. Some authors have suggested that VR1, also identified as the transient receptor potential cation channel subfamily V member 1 (TRPV1), should be classified as a cannabinoid 3-type receptor.

In the hippocampus CBR1s are mostly found in synapses that use γ-aminobutyric acid (GABA) as a neurotransmitter and mainly localized presynaptically, whereas CBR2s have a postsynaptic localization although their presence in presynaptic terminals cannot be excluded in some brain regions [43].

One of the physiological roles of CBR1s in the CNS is the regulation of synaptic transmission. eCBs attenuate presynaptic depolarization and subsequent neurotransmitter release [44]., thus mediating a retrograde regulation of this process. Upon activation, CBR1s repolarize the plasma membrane through the modulation of voltage-dependent ion channels that promote inward-rectifying K^+^ (Kir) and A-type K^+^ channels [45,46]. This is accomplished via the reduction in cAMP levels and PKA activation [47,48]. In addition, CBR1 activation can inhibit N-, P/Q- and L-type voltage-gated Ca^2+^ channels [49], thereby reducing Ca^2+^ influx and favoring repolarization of the presynaptic plasma membrane (see Figure 3).

Both CBR1 and CBR2 activation have been shown to favor phosphorylation and activation of the mitogen-activated protein (MAP) kinase cascade [50,51,52]. This metabolic cascade has an important role in the regulation of neuronal gene expression and synaptic plasticity. eCBs signaling is terminated by reuptake into both neurons and glial cells. Intracellular hydrolysis of AN and 2-AG is then catalyzed by the fatty acid amide hydrolase (FAAH) and monoacylglycerol lipase (MAGL) enzymes, respectively [53]. These lipid-derived molecules control the integration of signals generated by different neurotransmitters and synaptic neuromodulators that ultimately lead to diverse forms of homo-synaptic and hetero-synaptic meta-plasticity [54,55]. Thus, eCBs influence the flow of information that is key for the performance of many important high-order brain functions e.g., behavior [55,56,57].

Cannabinoids have been shown to alter the organization of the actin cytoskeleton in various cell types [58]. Njoo and collaborators highlighted the functional significance of CBR1 interactions with the Wiskott-Aldrich syndrome protein-family verprolin-homologous protein 1 (WAVE1)/SCAR1 complex [59]. These authors stated that the complex plays a key role in dynamically regulating the actin cytoskeleton in developing and adult neurons, thus contributing to the most salient functions of eCBs in the brain and spinal cord. Furthermore, they showed that cannabinoids structurally remodel dendritic spines by regulating the activity levels of WAVE1, and reported a novel function for WAVE1 in mediating inflammatory pain via structural and functional plasticity of spinal neurons [59].

Although CBR1s are mainly expressed presynaptically, several studies have indicated that CBR1 can be additionally localized in neuronal dendrites [60,61] colocalizing with the PSD protein PSD-95 in spines [59]. Rac1 activity decreases within minutes of CBR1 activation, limiting the conversion of G-actin to F-actin in dendritic spines of mature cortical neurons. This in turn produces a depletion of mature spines with the characteristic adult-type, elaborate mushroom morphology, which are believed to mediate increased synaptic efficacy such as observed in LTP [62]. In agreement with these findings, chronic treatment with the synthetic cannabinoid agonist WIN 55212-2 (WIN) reduced spine density in the nucleus accumbens (Nac) 24 h after the last injection [63]. Spiga and collaborators [64,65] also reported a decrease in spine density in the Nac (core) after a 1 h withdrawal period.

Activation of CBR1 in hippocampal neurons elicits a decrease in presynaptic F-actin and other cytoskeletal proteins, including ARPC2 and WASF1/WAVE1 that correlate with morphological changes (e.g., reduction in bouton size) [66], as seen at the postsynapse.

## 4. Cannabinoid Regulation of Synaptic Function and Compartmentalization

Neuronal circuits in hippocampus are fundamental in memory processes. Hippocampal pyramidal neurons perform important functions related to brain rhythms. Pyramidal cells are controlled by GABAergic interneurons, key for spatial memory formation. Potassium channels of the Kv7 type (KCNQ-Kv7) are characteristically located at the axon initial segment, where they modulate the membrane potential of the neuron and its cell firing. Oriens lacunosum moleculare interneurons, which are selectively active during theta oscillations, generate feedback inhibition of pyramidal neurons. In these somatostatin-positive interneurons, KCNQ-Kv7s are found predominantly in dendrites. Kv7 channel activity is upregulated following induction of presynaptic LTD. This depression of neuronal excitability involves eCB biosynthesis and depends on CBR1s, whereas eCBs acting directly on Kv7.2/3 channels, without participation of CBR1s, depress intrinsic neuronal excitability [67].

## 5. Cholinergic Signaling Contribution to Glutamatergic Receptor Compartmentalization

The paradigm excitatory neurotransmission in brain is mediated by glutamatergic synapses. These, in turn, are modulated by activation of presynaptic and postsynaptic neuronal nAChRs [68,69]. In the human brain, heteromeric α4β2 and homomeric α7 nAChRs constitute the most abundant subtypes of nAChRs, whereas other combinations are present in smaller numbers, and usually exhibit a more limited anatomical distribution, restricted to specific brain regions [70,71]. nAChRs are located on neurons where they regulate neurotransmitter release, cell excitability, and neuronal integration [72,73,74]. Activation of nAChRs can promote the release of glutamate (Glu), the main excitatory transmitter in mammalian brain, in a Ca^2+^-dependent manner. Presynaptic α4β2 or α7 nAChRs activation depolarizes hippocampal interneurons and indirectly affects the release of neurotransmitters like Glu and γ-aminobutyric acid (GABA) by activating voltage-gated Ca^2+^ channels [75]. Additionally, α7 nAChRs localized on brain glutamatergic terminals increases glutamate release upon activation [76] (Figure 1).

There is also evidence that the α4β2 and α7 subtypes of neuronal nAChRs contribute to changes in synaptic architecture, promoting glutamatergic synaptogenesis [77,78]. Postsynaptically located α7 nAChRs cause fast inward cationic currents in the neocortex and hippocampus. At this location α7 nAChRs have also been proposed to modulate synaptic potentiation independently of fast excitatory transmission [79] by recruiting GluA1 receptors from the surface pool of mobile extrasynaptic receptors and contributing to their stabilization in the spine via formation of receptor nanoclusters.. This stabilization requires functional PSD-95 scaffold protein family members that compartmentalize GluA1 macromolecules and help reduce their local mobility in conjunction with the actin submembrane meshwork. As a result, synapses enhance their signaling capacity, as reflected in the appearance of larger miniature excitatory postsynaptic currents (mEPSCs). This mechanism of GluA1 activation via α7 nAChRs is independent of fast excitatory transmission mediated by α-amino-3-hydroxy-5-methyl-4-isoxazolepropionic acid (AMPA) or N-Methyl-*D*-aspartic acid (NMDA) receptors (NMDARs, see Figure 1).

Nicotine can bind to nAChRs on dopaminergic neurons [80] or on nerve terminals of GABAergic and glutamatergic neurons that project on the dopaminergic neurons in the ventral tegmental area (VTA). Neurons from the VTA project to neurons in the nucleus accumbens (NAc) and are involved in the processing of reward signals that motivate behavior. Thus, activation of nAChRs induces the release of dopamine in the mesolimbic dopaminergic pathway, involving the cholinergic pathways in different brain reward mechanisms [81,82]. Dopaminergic signaling in the hippocampus, the amygdala and the prefrontal cortex help to create emotional associations with rewards. Hence, activation of nAChRs in these areas contributes to the regulation of dopaminergic homeostasis and the rewarding and psychostimulant effects of addictive drugs, including nicotine [83,84]. α4β2 nAChRs have been linked to nicotine reward since studies performed in rats showed that their blockage with antagonists significantly inhibited nicotine self-administration [85,86] while genetic deletion of α4 or β2 subunits prevented nicotine-induced increase in NAc dopamine levels [86,87,88]. Other nAChR subunits, including α3, α6, α7 and β3, have been reported to mediate dopamine release after nicotine induction [89].

Crosstalk between the dopaminergic and glutamatergic systems enables them to initiate and organize normal behavior [90]. NMDARs can act as a scaffold to recruit laterally diffusing dopamine D1 receptors (D1Rs) to spines. The activation of these NMDARs alters the topography and movement of D1Rs by trapping them in dendritic spines [91]. D1Rs selectively interact with the NR1 subunit of the NMDAR through its C-terminal tail to form dimeric hetero-complexes. Induction of LTP in the striatum requires activation of D1Rs since antagonizing these receptors blocks NMDAR–dependent LTP; while in the cortex, working memory is altered by this antagonism [92,93,94]. Activation of D1Rs via DA release, caused e.g., by cholinergic signaling activation, can then recruit D1R-NMDAR complexes in a regulated manner [95].

Cholinergic signaling pathways thus contribute to sub-compartmentalize glutamatergic neurotransmission, spatially restricting its sphere of action. More generally, the ubiquitous distribution of nicotinic receptors in brain enables them to regulate many important high-level cognitive functions such as attention, working memory, learning processes [96], cognitive flexibility [97] and social interactions [98]. They are also involved in addiction and dependence [81,82].

## 6. Cannabinoids and Nicotinic Receptors

In addition to the well characterized activation of CBRs, cannabinoids also exert receptor-independent and lipid bilayer-mediated actions on many membrane proteins, enzymes, and transporters [99,100]. There is evidence of overlapping distribution of CBRs and nAChRs in many brain structures [101]. At the level of brain reward pathways, for instance, the endocannabinoid and nicotinic cholinergic systems interact bidirectionally, having an important role in the modulation of drug dependence [101]. In animal studies, the primary psychoactive ingredients in cannabis and tobacco, delta-9-tetrahydrocannabinol (Δ9-THC) and nicotine, respectively, show overlapping pharmacological effects, including induction of antinociception, hypothermia, rewarding effects, dependence, and impairment of locomotion [102,103,104,105,106,107]. Additionally, Δ9-THC and nicotine potentiate their anxiolytic effects in a synergistic manner when administered together at sub-threshold doses [108,109,110,111]. Chronic administration of nicotine in rats increases AN levels in the limbic forebrain and of both AN and 2-AG in the brainstem [89]. Reciprocally, Δ9-THC and synthetic cannabinoids modulate cholinergic neurotransmission in the hippocampus [112,113,114], supporting the notion that nicotinic and endocannabinoid systems interact in the brain reward pathway. This information opens a window for therapeutic intervention in nicotine and cannabinoid dependence [115,116].

Other experimental demonstrations of how cannabinoids and nicotinic receptors interact can be found in the direct actions of endocannabinoids on α7 nAChR in *Xenopus* oocytes [117,118]. In this experimental model system, AN reversibly inhibited nicotine-induced currents in a concentration-dependent manner without significantly affecting its half maximal effective concentration (EC50) value, indicating that AN acts as a noncompetitive antagonist on α7 nAChRs. In agreement with these observations, Frey and coworkers reported that AN does not inhibit specific [^3^H]nicotine binding in human frontal cortex membranes [119], providing supporting evidence for the noncompetitive action of AN on nicotinic receptors. A direct inhibitory action of AN on nAChRs is suggested by the lack of effect on CBR1, CBR2, or enzymes involved in AN metabolism. It must be noted however that the synthetic cannabinoids WIN55,212-2 and THC do not alter α7 nAChR function. Instead, AN markedly inhibits the peak amplitudes of α4β2 nAChR-mediated currents to about 30% of control levels in SH-EP1 cells [120]. In addition, studies performed on myenteric neurons have shown that AN can reduce the amplitudes of nicotine-induced inward currents [99,121].

The lipophilic nature of eCBs facilitates their incorporation into biological membranes, and their accumulation has been shown to modulate the action of alcohol and volatile anesthetics on α7 nAChRs [122,123]. These pharmacological effects suggest that CBs may alter the bulk physicochemical properties of the plasma membrane. Thus, the two types of endogenous neurotransmitters, eCB and acetylcholine can directly or indirectly (following binding to hydrophobic sites on the receptor surfaces) modulate the function of these receptors in brain and exert modulatory crossover effects on each other.

In the activated spine, Ca^2+^ entry through postsynaptic α7 nAChRs [70] or NMDA receptors and/or voltage-gated Ca^2+^ channels [124] can promote the activation of multiple signaling pathways with specific spatio-temporal patterns that orchestrate and regulate different aspects of cytoskeletal dynamics in the stimulated spines. Within the spine, Ca^2+^ binds to calmodulin (CaM), a Ca^2+^-binding protein which subsequently activates Ca^2+^/CaM-dependent kinases and phosphatases such as CaMKII and calcineurin [125,126]. CaMKII activates small GTPases and these in turn further modulate several downstream kinases [127] that have the ability to activate many ABPs, including Cofilin and Arp2/3, two proteins that play essential roles in actin remodeling [128,129]. Thus cannabinoids, through rac1/WAVE 1 modulation and α7 nAChR activation, contribute to actin structural remodeling of dendritic spines. Whereas activation of Rac1/WAVE1 induces a depletion of mushroom type spines, α7 nAChR activation contributes to the formation and maturation of dendritic spines (Figure 1).

## 7. Importance of Lipids in Dendritic Spine Compartmentalization

Lipids play a central role in the formation and shaping of membranes [130,131]. More than 40 different lipids modulate signaling and dynamically influence membrane geometry of synapses [132,133,134,135]. As in other biological membranes, the major classes of lipids found at synaptic plasma membranes are cholesterol, sphingolipids and glycerol backbone-based phospholipids [136]. Of these, cholesterol and saturated sphingolipids and phospholipids are particularly enriched in membrane domains [137], and the cholesterol concentration in brain accounts for ~25% of the human body’s total content.

The neutral lipid cholesterol plays a key role in receptor clustering and compartmentalization [77,79]. The amount of cholesterol and its topographical distribution at the plasma membrane have a profound influence on the physicochemical properties of the plasmalemma, and these, in turn, modulate the functional activity of the synapse. The most general effect of cholesterol is exerted on the physical properties of the bulk membrane. Membrane cholesterol concentrations above a certain threshold level promote the formation of liquid-ordered (Lo) domains [138] that are thicker than the rest of the membrane and exhibit unique biophysical properties. These ordered lipid nanodomains (“lipid rafts”) mediate trafficking, signal transduction, and membrane protein activity in essentially all eukaryotic plasma membranes [139].

Cholesterol increases membrane rigidity, thickness and tension that influences cell signaling and domain formation [140,141]. The concept of lipid domains entails not only the existence of lateral heterogeneities in the plane of the membrane, but also the dynamic exchange of molecular components within and between compartments. For instance cholesterol flip-flops between the outer and inner leaflets of the membrane bilayer, and also diffuses within a given liquid-ordered lipid domain and between different lipid domains in inter-domain exchange processes which are quite heterogeneous, differing considerably in their kinetic parameters [142].

At the synapse, cholesterol interacts with several neurotransmitter receptors through consensus linear binding sequences like the so-called cholesterol recognition/interaction amino acid consensus motifs (CRAC and its mirror image CARC [143]. These consensus domains have been proposed to facilitate membrane protein incorporation into cholesterol-rich domains in a great variety of membrane proteins, including the superfamily of pentameric ligand-gated ion channels (pLGICs) and the superfamily of G-protein coupled receptors (GPCRs). The prototypic LGIC, the nAChR, exhibits a CRAC motif adjacent to the transmembrane helix M1, and a CARC sequence on the M4-facing surface of M1 adjacent to one of the proposed cholesterol-binding cavities [143,144]. Likewise, the transmembrane helix 7 of human cannabinoid receptor 1 (CBR1) displays a CRAC sequence [145]. A cholesterol molecule was recently identified in a crystal structure [146] and a cryo-electron microscopy (EM) structure [147] of the CBR1.

Sphingolipids participate as plasma membrane lipids and signaling molecules such as ceramide, sphingosine, and sphingosine-1-phosphate that are produced after the metabolism of plasma membrane sphingolipids [148]. Among sphingolipids, sphingomyelins are enriched in brain membranes. The elimination of dendritic spines upon reduction of cholesterol and sphingomyelin levels was described almost 20 years ago [149], highlighting the importance of these lipids for neuronal communication. Ceramide promotes spine maturation by contributing to the transformation of dendritic filopodia to mature spines [150].

Phosphoinositides (PIPs) are important players in postsynaptic excitability since they have an exceptional high rate of metabolic turnover and compartmentalization [151]. There are multiple enzymes at dendritic spines that interconvert different PIPs contributing to the dynamic role of lipids in the plasma membrane. Phosphatidylinositol (4,5) diphosphate (PIP2) is converted to phosphatidylinositol (3,4,5) triphosphate (PIP3) by phosphatidylinositol-4,5-bisphosphate 3-kinase. PIP3 content at the spines is higher than that in dendritic shafts under basal conditions. Furthermore, upon glutamate stimulation PIP3 redistributes contributing to the formation of fine spinules projecting from spines [152,153]. Additionally, PIP3, because of its capacity regulate the activity of multiple Rho GTPase effectors [154], has also been implicated in the interaction of membrane-cytoskeleton crosstalk at spines, and is able to regulate the Akt-mTOR pathway to participate in dendritic spine morphogenesis [155,156]. The PIP2-clustering molecule myristoylated alanine-rich C kinase substrate (MARCKS) reversibly sequesters PIP2 on the plasma membrane, upon local increases in intracellular calcium [157]. MARCKs contributes to spine morphogenesis by promoting the transition from immature dendritic spines to larger and more stable mushroom-shaped spines by controlling actin cytoskeleton [158]. Moreover, association of MARCKS to cholesterol at the membrane is necessary for its ability to crosslink F-actin [158]. In addition, PIP3 contributes to the accumulation of PSD-95 at spines whereas conversion of PIP2 by phospholipase C favors synaptic actin depolymerization and PSD-95 degradation, thus contributing to spine remodeling [159].

Among other factors, actin dynamics is modulated by membrane lipids. A reduction in membrane cholesterol levels produces a rapid collapse of spine morphological integrity associated with redistribution of F-actin from the spine proper to the dendritic shaft [149]. Sphingolipids also play a relevant role in the spine plasma membrane-actin cytoskeleton crosstalk. Sphingomyelins modulate membrane binding and activity of the Rho GTPases, key regulators of the actin cytoskeleton in the synapse. Accumulation of sphingomyelins at postsynaptic membranes, as observed in a Niemann-Pick disease type A mouse model defective in acid sphingomyelinase, induces a reduction of metabotropic glutamate receptors that impairs the membrane attachment of RhoA and its effectors ROCK (RhoA kinase) and profilin IIa. This impairment results in the diminution of F-actin content, ultimately reducing spine number and size [160]. The conversion of sphingomyelin to ceramide at the plasma membrane is catalyzed by neutral sphingomyelinase-2 [161]. This enzyme can, in turn, modulate spine actin cytoskeleton. Activation of the neutral sphingomyelinase restores F-actin content of dendritic spines by enhancing the RhoA pathway in mice defective of the acid sphingomyelinase, which present high sphingomyelin synaptic levels [160]. Conversely, inhibition of the sphingomyelinase decreases the abnormally high levels of F-actin in spines of neurons in mice lacking Wiskott–Aldrich syndrome protein interacting protein (WIP) [162]. Thus, through various protein and lipid interactions, actin promotes compartmentalization of the plasma membrane and has an important role in the modulation of neuronal strength.

### Impact of the Lipid Microenvironment on nAChRs and CBRs

The best documented example of the influence that the lipid microenvironment and cholesterol exert on the topography and function of a neurotransmitter receptor is provided by the paradigm rapid LGIC, the nAChR (see reviews in [163,164]). Changes in cholesterol levels alter the translational mobility of the receptor in the plane of the plasma membrane, as measured by fluorescence recovery after photobleaching and fluorescence correlation spectroscopy [165] and single-molecule localization microscopy [166,167]. Cell-surface trafficking of nAChRs is dependent on cholesterol metabolism [168,169]. Pharmacological long-term inhibition of cholesterol biosynthesis by the statin lovastatin differentially augments cell-surface levels of α4β2 and α7 nAChRs in neurites and soma of rat hippocampal neurons [169]. Misbalances in brain cholesterol homeostasis affect cholinergic signaling involving α4β2 and α7 nAChRs and indirectly impact on the number and distribution of other neuroreceptors at dendritic spines, with important consequences for brain function. Sphingolipids are also necessary for nAChR export in the early secretory pathways [170]. Thus, any modification in sphingolipid levels will impact on nAChR expression.

CBRs are also influenced by the lipid composition of the cell membrane [171]. In particular, cholesterol has been reported to negatively modulate the activity of CBR1 in nerve cells [172] and a specific cholesterol binding site has been described in the CBR1 molecule [145]. Likewise, cholesterol has recently been reported to increase the basal activation levels of the CBR2 receptor by exerting an allosteric effect on the intracellular regions of the receptor [171]. Considering the retrograde regulatory effects of CBRs on neurotransmitter release [44] lipid compartmentalization by membrane cholesterol is of considerable importance as it provides additional regulatory check points of neurotransmitter receptor function.

Furthermore, the lipophilic endogenous neurotransmitter AN exhibits selectivity for cholesterol and ceramide amongst membrane lipids. Through the establishment of a hydrogen bond, AN forms a molecular complex with cholesterol inside the plasma membrane, and has been proposed to interact with CBR1 via the cholesterol-recognition motifs CRAC or CARC [173]. AN binding to ceramide blocks the degradation pathway of both lipids [173].

## 8. Contribution of Actin Dynamics to the Compartmentalization of the Dendritic Spine

The eukaryotic plasma membrane is connected to the intracellular milieu via many adapter proteins [174]. The plasma membrane domains are associated with intracellular structures, and the submembrane cytoskeletal meshwork is one of the important elements that regulate cell mechanics and morphology, contributing to delimiting the extension of membrane domains. The actin cytoskeleton modulates plasma membrane dynamics and introduces barriers to the diffusion of membrane proteins, with the formation of micrometer-sized corrals and smaller domains where membrane proteins can experience anomalous diffusion [175,176,177,178]. These actin meshwork-driven compartments play an important role in the dendritic spine. For instance, disruption of PDZ-containing scaffolds (PDZ domains are protein–protein interaction modules that recognize specific C-terminal sequences) or of actin filaments in chick ciliary ganglion neurons has been shown to increase the mobility of α7 nAChRs [179]. In contrast, making the sub-membrane cortical actin network more stable by cholesterol depletion [180] reduces receptor mobility. Cholesterol depletion diminishes nAChR mobility at the cell surface; mobility can be partially restored upon treatment with Latrunculin A [165].

Actin polymerization and depolymerization dynamics are active modifiers of presynaptic morphology, with important functional implications [181,182]. Thus, the actin cytoskeleton provides the adequate scaffold for synaptic vesicles and the active zone [183,184]. Changes in the size of presynaptic terminals have been correlated with the postsynaptic response [185,186,187] and thus with synapse strength [187,188,189,190]. At the postsynapse, actin filament dynamics provide a framework for the formation of transient protrusions and modifications of dendritic spine morphology by interacting with a variety of actin-binding proteins (ABPs) [191]. These ABPs are important in actin dynamics, including actin polymerization-depolymerization, nucleation, branching, capping, cross-linking and trafficking [192].

## 9. Nanodomain Cluster Organization Emerges as a Common Organizing Principle at CNS Synapses

Neuronal activity regulates receptor fluxes at the PSD through the interaction of receptors with scaffolding molecules and lipids [193]. The PSD scaffolding proteins are distributed heterogeneously and form nanodomains within the synapses, termed sub-synaptic domains (SSD) [194]. The characteristic output of LTP induction, spine enlargement, is paralleled by the increase in the number of PSD-95 (an SSD-resident protein) copies [195]. This augmentation in PSD-95 biosynthesis occurs within hours of LTP induction [196].

During development, proteins organized in nanoclusters at the pre- and postsynapse control the differentiation of the dendritic spine. The cholesterol-binding protein TSPAN5, which belongs to the tetraspanin superfamily, is localized postsynaptically in pyramidal excitatory neurons, and is the master controller of spine maturation. Control is exerted by promoting the clustering of the postsynaptic molecule neuroligin-1 at the spine surface [197], which in turn recognizes and binds to presynaptic neurexins. TSPAN5 knockout mice have an extremely low number of spines, reinforcing the view that this tetraspanin plays a key role in excitatory synapse maturation.

Neurotransmitter receptors are also organized into SSDs at the postsynaptic plasma membrane [198]. Neurotransmitter receptor domains localized in SSD domains exhibit diffusion-limited lateral mobility relative to receptors localized in non-synaptic areas, and the two pools are in active exchange [199]. Receptors in either pool are removed from the plasma membrane via metabolic turnover [200]. The maintenance of a homeostatic balance between these two mechanisms determines the number of active receptors at the postsynaptic membrane.

**Figure 4:**
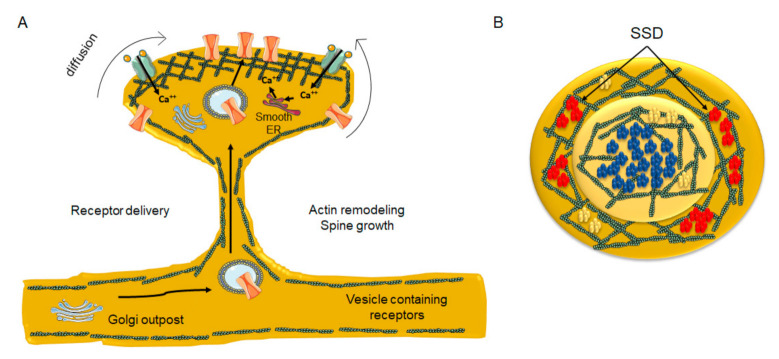
Diagrammatic depiction of dendritic spine remodeling during synaptic plasticity and associated neurotransmitter receptor clustering in nanodomains. (**A**) remodeling of the submembrane actin meshwork and incorporation of newly synthesized/laterally diffusing receptors from non-active areas of the spine head and neck into the PSD area, thereby increasing neurotransmitter receptor number at the site of contact with presynaptic boutons. At the crest of the spine receptors become entrapped by actin corrals, which might also present a lipid composition distinct from the bulk lipid bilayer. Golgi outpost in the dendrite and satellite Golgi outposts are found in the spine head, as well as smooth ER outposts. (**B**) Top view of the PSD. NMDARs (blue) are predominantly located at the center of the PSD in a single nanocluster, whereas AMPARs (red) are segregated into several nanodomains (sub-synaptic domains, SSD) surrounding the central NMDAR nanodomain. In contrast, mGluR5 (yellow) are aggregated into small clusters or homogeneously distributed at the PSD.

At excitatory glutamatergic synapses, the lateral exchange of receptors between extrasynaptic and synaptic areas is responsible for neuronal plasticity [193,201,202]. In the CNS, endocytosis occurs predominantly, if not exclusively, in extrasynaptic areas [203,204,205], and hence receptors have to diffuse out of the PSD to be internalized [206]. In turn, mobilization of receptors from the spine shaft to the PSD partly depends on the morphology of the spine. As the spine neck diameter diminishes, it is energetically and mechanically more costly to transport endosomal vesicles from the ER outposts in the dendrite to the PSD through the spine actin meshwork [207]. Dendritic ER and Golgi outposts are well documented in rodent hippocampal neurons, particularly at dendritic branching points [208] and high-pressure freezing fixation (without chemical fixation) combined with transmission electron microscopy has revealed smooth ER and Golgi outposts inside dendritic spines [209] (Figure 4).

Formation of cell-surface receptor nanoclusters is favored when stabilizing interactions preclude lateral diffusion of individual receptor molecules [210,211]. Upon disruption of the cytoskeleton, α7 nAChR mobility and cell surface expression are hampered, but not the receptors’ ability to form clusters, suggesting tethering mechanisms [212].

Perisomatic GABAergic terminals from CBR1-positive basket cell interneurons produce stronger endocannabinoid-mediated synaptic plasticity than that mediated by dendritic synapses [213]. Consistent with these observations, studies combining stochastic optical reconstruction microscopy (STORM) superresolution optical microscopy with whole-cell patch-clamp electrophysiological recordings found that GABAergic interneurons projecting axon terminals on perisomatic synapses possess larger synaptic boutons, harboring higher numbers of CBR1s, higher receptor/effector ratio, and more complex active zones than dendritic spine-projecting interneurons, whereas nanoclusters of the presynaptic active zone-resident protein Bassoon were more abundant in dendritic synapses [214]. The CBR1/Bassoon ratio was 46% higher within 100 nm of the Bassoon nanocluster edges in perisomatic boutons than in dendritic nerve endings. These authors also found that chronic Δ9-THC administration, which diminishes cannabinoid efficacy on GABA release, markedly downregulated CBR1s. Complete receptor recovery occurred only weeks after the cessation of Δ9-THC treatment. Dudok and coworkers hypothesized that the nanoscale receptor/effector ratio and coupling distance, rather than the number of receptors, determines cannabinoid signaling and neurotransmitter release [214].

Recently, Goncalves and coworkers showed that NMDARs form a single SSD located mainly at the center of the PSD, whereas AMPARs were segregated into several SSDs surrounding the NMDAR central nanocluster and mGluR5 presents a more diffuse distribution [215]. This observation is in agreement with the notion that NMDARs are less mobile than AMPARs at mature synapses [202,216] (Figure 4B). As for CBRs, CBR1 colocalizes with PSD-95 in spines [59]. The predominantly presynaptic CBR1s have been reported to be uniformly distributed at the nanoscale level [214]. However, as is the case with the nAChR, changes in cholesterol/sphingolipid ratio and actin stability can alter the distribution of CBR1s. In addition, a reduction in the number of cell-surface CBR1s via endocytic internalization [47,217] or through the action of β-arrestins [218,219] can be induced after long agonist exposure. Binding of β-arrestins uncouples G-proteins and stimulates receptor internalization and β-arrestin mediated signaling [218,220].

## 10. Concluding Remarks

Compartmentalization provides the plasma membrane with discrete 2-D platforms where biochemical reactions or signaling mechanisms can be simultaneously executed with maximal efficacy, without interference from competing processes. The dendritic spine, a highly differentiated subcellar compartment distinct from the rest of the neuronal membrane, maximizes its operational capacity by incorporating nanodomain compartments at its surface. One of the mechanisms exploited by the dendritic spine to parcel out its cell-surface membrane is through the formation of reversible biological barriers via the actin meshwork. This sub-membrane network dynamically interacts with the plasmalemma, creating barriers that transiently hinder protein translational diffusion, thereby secluding molecules from the rest of the bilayer and transiently retaining them in nanodomain compartments.

Advances in the field of dendritic spine compartmentalization should provide important clues about the mechanisms involved in the regulation and function of neurotransmitter receptors and their interplay with scaffolding proteins, lipids, and enzymes at brain synapses in health and disease.
